# A new leaf-mining moth from New Zealand, *Sabulopteryxbotanica* sp. nov. (Lepidoptera, Gracillariidae, Gracillariinae), feeding on the rare endemic shrub *Teucriumparvifolium* (Lamiaceae), with a revised checklist of New Zealand Gracillariidae

**DOI:** 10.3897/zookeys.865.34265

**Published:** 2019-07-22

**Authors:** Robert J.B. Hoare, Brian H. Patrick, Thomas R. Buckley

**Affiliations:** 1 New Zealand Arthropod Collection (NZAC), Manaaki Whenua–Landcare Research, Private Bag 92170, Auckland, New Zealand New Zealand Arthropod Collection Auckland New Zealand; 2 Wildlands Consultants Ltd, PO Box 9276, Tower Junction, Christchurch 8149, New Zealand Wildlands Consultants Ltd Christchurch New Zealand; 3 School of Biological Sciences, The University of Auckland, Private Bag 92019, Auckland, New Zealand The University of Auckland Auckland New Zealand

**Keywords:** New species, taxonomy, New Zealand, leaf miners, herbarium, checklist

## Abstract

*Sabulopteryxbotanica* Hoare & Patrick, **sp. nov.** (Lepidoptera, Gracillariidae, Gracillariinae) is described as a new species from New Zealand. It is regarded as endemic, and represents the first record of its genus from the southern hemisphere. Though diverging in some morphological features from previously described species, it is placed in genus *Sabulopteryx* Triberti, based on wing venation, abdominal characters, male and female genitalia and hostplant choice; this placement is supported by phylogenetic analysis based on the COI mitochondrial gene. The life history is described: the larva is an underside leaf-miner on the endemic divaricating shrub *Teucriumparvifolium* (Lamiaceae), and exits the mine to pupate in a cocoon in a folded leaf of the host plant. The remarkable history of the discovery and rediscovery of this moth is discussed: for many years it was only known from a single sap-feeding larva found in a leaf-mine in a pressed herbarium specimen of the host. The adult was discovered by BHP in Christchurch Botanic Gardens in 2013. Most distribution records of the moth come from a recent search for mines and cocoons on herbarium specimens of *T.parvifolium*. *Sabulopteryxbotanica* has high conservation status, and is regarded as ‘Nationally Vulnerable’ according to the New Zealand Department of Conservation threat classification system, based on the rarity and declining status of its host plant. However, the presence of apparently thriving populations of *S.botanica* on cultivated plants of *T.parvifolium*, especially at the type locality, Christchurch Botanic Gardens, suggests that encouraging cultivation of the plant could greatly improve the conservation status of the moth. A revised checklist of New Zealand Gracillariidae is presented, assigning all species to the currently recognised subfamilies. The Australian *Macarostolaida* (Meyrick, 1880) is newly recorded from New Zealand (Auckland), where it is established on *Eucalyptus*.

## Introduction

New Zealand has a relatively depauperate fauna of the leaf-mining moth family Gracillariidae: revision and further field work can be expected to increase the number of species, but the fauna is probably relatively well known and genuinely species-poor. Dugdale (1988: 70–72) listed 21 named species, and mentioned two unnamed species. Hoare (2001) added two further adventive species, *Dialecticascalariella* (Zeller, 1850) and ‘Acrocercops’ laciniella (Meyrick, 1880). The Australian *Macarostolaida* (Meyrick, 1880) was discovered established in Auckland and Northland on planted *Eucalyptus* in January 2019. A further two unnamed endemic species have also been recognised since Dugdale’s catalogue, one of which is described in this paper. From this total of 28 species, 22 are endemic to New Zealand, five are adventive from Australia, and one (*Phyllonoryctermessaniella* (Zeller, 1846)) is adventive from Europe. Kawahara et al. (2017) recently presented a molecular phylogeny and revised subfamily classification of world Gracillariidae, recognising eight monophyletic subfamilies. In their recent checklist of neotropical Gracillariidae, De Prins et al. (2019) largely followed this revised classification, but reduced Parnornichinae to the status of a tribe (Parornichini) within Gracillariinae and regarded Oecophyllembiini and Marmarini as tribes within an expanded Phyllocnistinae. The classification of De Prins et al. (2019) is consistent with the phylogeny of Kawahara et al. (2017), but the change in ranks was introduced without explicit justification. We note that the nodes supporting the more narrowly defined subfamilies of Kawahara et al. (2017: fig. 2) have stronger bootstrap support values than those supporting each of the expanded subfamilies of De Prins et al. (2019), indicating that the former classification is likely to be more stable, as well as being simpler. We therefore follow the Kawahara et al. (2017) classification here.

Given these recent changes and discoveries, we present an updated New Zealand Gracillariidae checklist here (Appendix 1) and assign the named species as far as possible to the newly defined subfamilies; further changes can be expected once the fauna is revised.

**Figure 1.**
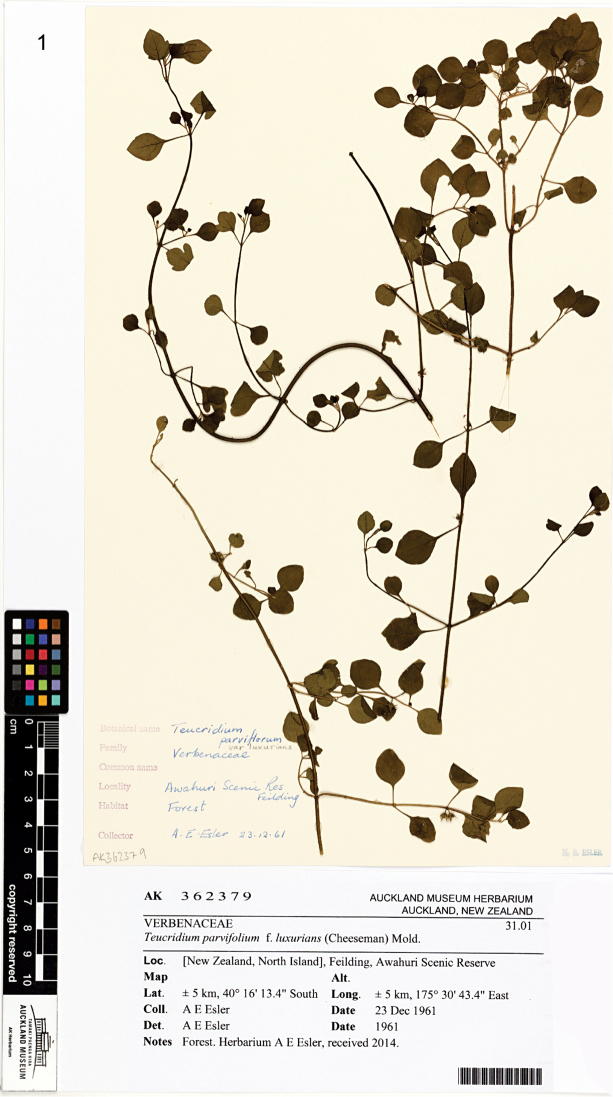
*Teucriumparvifolium*. The specimen from Awahuri Reserve, Feilding, collected by AE Esler on 23 Dec 1961, on which the first mine of *Sabulopteryxbotanica* was detected (Auckland Museum AK362379). Photograph courtesy of Ewen Cameron, Auckland Museum herbarium: the sheet label has been enlarged in this reproduction relative to the specimen.

The new species described here was first detected as an early instar larva pressed inside its linear leaf-mine in a herbarium specimen of *Teucriumparvifolium* (Hook. f.) Kattari & Salmaki (Lamiaceae). This larva was collected in the southern North Island at Awahuri Reserve near Feilding WI by the botanist Alan E. Esler on 23 December 1961; it was shown to H. Donner and C. Wilkinson when they were revising the New Zealand Nepticulidae fauna (Donner and Wilkinson 1989). Those authors recognised that the larva was not a nepticulid based on the lack of a spinneret and the lack of anal rods, but they did not assign it to another family. Following this discovery, BHP searched unsuccessfully for mines on *T.parvifolium* at Trotter’s Gorge DN, where the plant is common (Donner and Wilkinson 1989). The Awahuri Reserve specimen is still in the ethanol collection in NZAC, and was examined in 1998 by RJBH, who determined it as a sap-feeding early instar gracillariid larva. The associated pressed specimen of *Teucriumparvifolium* from the Esler collection is now in the Auckland Museum herbarium (AK362379; Fig. 1) and was examined by RJBH in June 2018: no further mines were found on this specimen.

In September 1999, RJBH searched unsuccessfully for *Teucrium* plants at Awahuri Reserve in company with Grace Hall (NZAC). In December 2000, flowering *Teucrium* plants were located at Carters Scenic Reserve near Carterton, WA, but no mines or other damage were discovered. The miner was finally rediscovered on 31 January 2004, when Nicholas Martin collected a sprig of *T.parvifolium* at Paengaroa Scenic Reserve, Mataroa, near Taihape RI, with two mined leaves. He discovered a further mine in a *Teucrium* leaf at Price’s Valley, Banks Peninsula MC on 17 August 2005. These pressed mines are in NZAC; no moths were reared.

The matter rested there until 29 January 2013, when BHP disturbed an adult gracillariid from a plant of *Teucriumparvifolium* during a lunchtime walk in the Christchurch Botanic Gardens (Patrick 2018). Further searches revealed the moth to be common amongst its host, and mines and larvae were soon discovered and reared through, confirming that this was the mystery species known for over 50 years only from pressed leaves. Later, the moth was discovered on indigenous remnants of the host plant on Rakaia Island, south-west of Christchurch (Patrick and Grove 2015), rediscovered in Price’s Valley, and also found on *Teucrium* amongst the restored native vegetation planted by botanist Carol Jensen at Kennedy’s Bush, near Halswell Quarry, Christchurch. Searches of herbarium specimens in the Allan Herbarium (Lincoln) and the Auckland Museum herbarium in 2018 revealed a number of further leaf-mines from almost throughout the plant’s range (see Distribution and Remarks under species description below).

The moth is described as new here and assigned to the genus *Sabulopteryx* Triberti, 1985, in the subfamily Gracillariinae (in the sense of Kawahara et al. 2017). *Sabulopteryx* was described as a subgenus of *Aspilapteryx* Spuler, 1910, but removed from synonymy by Pereira et al. (2019) (see under Systematic placement, below). This is the first record of the genus from the southern hemisphere.

## Materials and methods

Specimens were reared for this study by enclosing twigs with mined leaves of the host plant in plastic containers lined with absorbent paper. Leaves of *Teucriumparvifolium* dry out readily when picked and care must be taken to maintain sufficient moisture while avoiding mould. Genitalia and wing venation preparations followed the methods outlined by Hoare (2000) for Nepticulidae, except that the abdominal cuticle was opened up along the side by means of pulling with fine forceps, and a 2% solution of acid fuchsin in 70% ethanol was substituted for the acid fuchsin-azophloxin stain described in that paper. Only the abdominal cuticle and wings and not the genitalia were stained with acid fuchsin; male and female genitalia were stained with Chlorazol Black E. Terminology for the male and female genitalia follows Kumata (1982) and Triberti (1985). Larvae were preserved and examined in 75% ethanol. Pupal exuviae were slide-mounted in Euparal.

All herbarium sheets of *Teucriumparvifolium* in the Auckland Museum herbarium (Auckland) and the Allan Herbarium (Manaaki Whenua – Landcare Research, Lincoln) were searched for preserved leaf-mines by RJBH in June 2018.

All specimens used for drafting the description of the new species are held in NZAC (New Zealand Arthropod Collection, Manaaki Whenua–Landcare Research, Auckland, New Zealand). Additional specimens, including the first adults found of the new species, are in **BPNZ** (Brian Patrick collection, Christchurch, New Zealand).

Plant names for New Zealand plants follow the New Zealand Plant Conservation Network website (NZPCN 2019); readers should refer to this site for authorship of the native host plants listed in Appendix 1; authorities for introduced plants are given in the text. Two-letter area codes for regions of New Zealand are as defined by Crosby et al. (1998).

### Molecular systematics

DNA was extracted from legs of two paratype specimens of *Sabulopteryxbotanica* using the Qiagen DNeasy Blood & Tissue Kit. The 5’ region of the mitochondrial cytochrome c oxidase subunit I (COI) gene was amplified using the primers described by Folmer et al. (1994). Polymerase chain reactions (PCR) were performed using 25 µL volumes containing 2 µL of genomic DNA extract, 2.0 µL PCR Buffer with MgCl2 (Roche, USA), 2.0 µL 2mM dNTP, 0.8 µL BSA (10mg/mL), 0.5 µL of 10 µM each primer, 1.0 µL of Fast start Taq DNA polymerase (Roche, USA). Thermal cycling conditions were an initial denaturation at 95° for 4 minutes, the 38 cycles of 94° for 45 seconds, 55° for 45 seconds and 72° for 45 seconds. This was followed by a final extension of 72° for 5 minutes. PCR products were purified using the Xterminator Purification Kit (Thermo Fisher) and cycle sequenced used BigDye Terminator Version 3.1 (Applied Biosystems). Cycle sequencing conditions followed Platt et al. (2007). The products were run on a 3100-Avant Genetic Analyzer (Applied Biosystems). The two resulting COI sequences have been submitted to Genbank with accession numbers MK797749 and MK797750.

DNA sequences were edited and aligned in Geneious v. 10.2.6 (Kearse et al. 2012). We downloaded the COI sequences from the Gracillariidae phylogeny by Huemer et al. (2016) from NCBI. From Genbank we also downloaded COI sequences for *Aspilapteryxmultipunctella* (Chrétien, 1917) (KX042619), *Sabulopteryxlimosella* (Duponchel, 1844) (KP253447) and *S.inquinata* (Triberti, 1985) (KP150259). Following Huemer et al. (2016) we rooted the phylogenies using *Anthophilafabriciana*(Linnaeus, 1767) (Choreutidae). PCR amplification of the histone subunit 3 gene region, included in the Huemer et al. (2016) study, was unsuccessful.

Phylogenetic relationships were reconstructed using MrBayes v. 3.2.6 (Huelsenbeck and Ronquist 2001). We used the GTR+I+Γ model with the following prior distributions; unconstrained branch lengths (gamma parameter = 1.0), among-site rate variation (exponential parameter = 10), proportion of invariable sites (uniform 10 – 1). Each MCMC analysis was run with four chains, five million generations, thinning interval of 1,000, heating temperature of 0.2, and a burnin of 1 million. This analysis was repeated five times to ensure convergence. The MCMC output was summarised in Geneious v. 10.2.6.

## Taxonomy

### Sabulopteryx botanica

Taxon classificationAnimaliaLepidopteraGracillariidae

Hoare & Patrick sp. nov.

653f4624-f62e-405a-aa81-11f8c96ad8f1

http://zoobank.org/AE827276-BA17-4BA6-83BC-A62912E77CF2

Figs 2–3 , 4–6 , 7–11 , 12 , 14–15 , 16–17

#### Material examined.

***Holotype***: NEW ZEALAND • ♂; Mid Canterbury [MC], Christchurch Botanic Gardens; 43°31.8'S, 172°37.2'E; emg. 21 Apr. 2014; R.J.B. Hoare, B.H. Patrick leg.; larva in leaf-fold on *Teucridiumparvifolium* 31 Mar. 2014; NZAC.

***Paratypes***: NEW ZEALAND • 1 ♀; same collection data as holotype; emg. 24 Apr. 2014; NZAC • 3 ♂♂; MC, Christchurch Botanic Gardens; 31 Mar. 2014; R.J.B. Hoare, B.H. Patrick leg.; beaten from *Teucriumparvifolium* [as *Teucridium* on labels]; NZAC • 2 ♀♀; same collecting data as preceding; ♀ genitalia on slides NZAC Grac. 2, NZAC Grac. 4; NZAC • 4 ♂♂; MC, Christchurch, Kennedy’s Bush Rd, Jensen property; 29 Mar. 2014; R.J.B. Hoare, B.H. Patrick leg.; on and around *Teucriumparvifolium*; ♂ genitalia and wings on slide NZAC Grac. 3; NZAC • 1 ♂; MC, Banks Peninsula, Prices Valley; 1 Apr. 2014, R.J.B. Hoare, B.H. Patrick leg.; beaten from *Teucriumparvifolium*; ♂ genitalia on slide NZAC Grac. 1; NZAC.

#### Diagnosis.

*Sabulopteryxbotanica* is distinctive amongst New Zealand gracillariids in its combination of small size (wingspan 10 mm or less) and yellow-ochreous black-speckled forewings. It is perhaps most similar to *Caloptiliaselenitis* (Meyrick, 1909), but this species has the centre of the vertex white and has three white spots along the forewing dorsum (there is no white on the vertex or forewing in *S.botanica*). In the male genitalia, the paired processes on the dorsum of the valva are diagnostic, and in the female, the deep invaginations of the S7–S8 intersegmental membrane are characteristic.

#### Description.

**Figures 2, 3.**
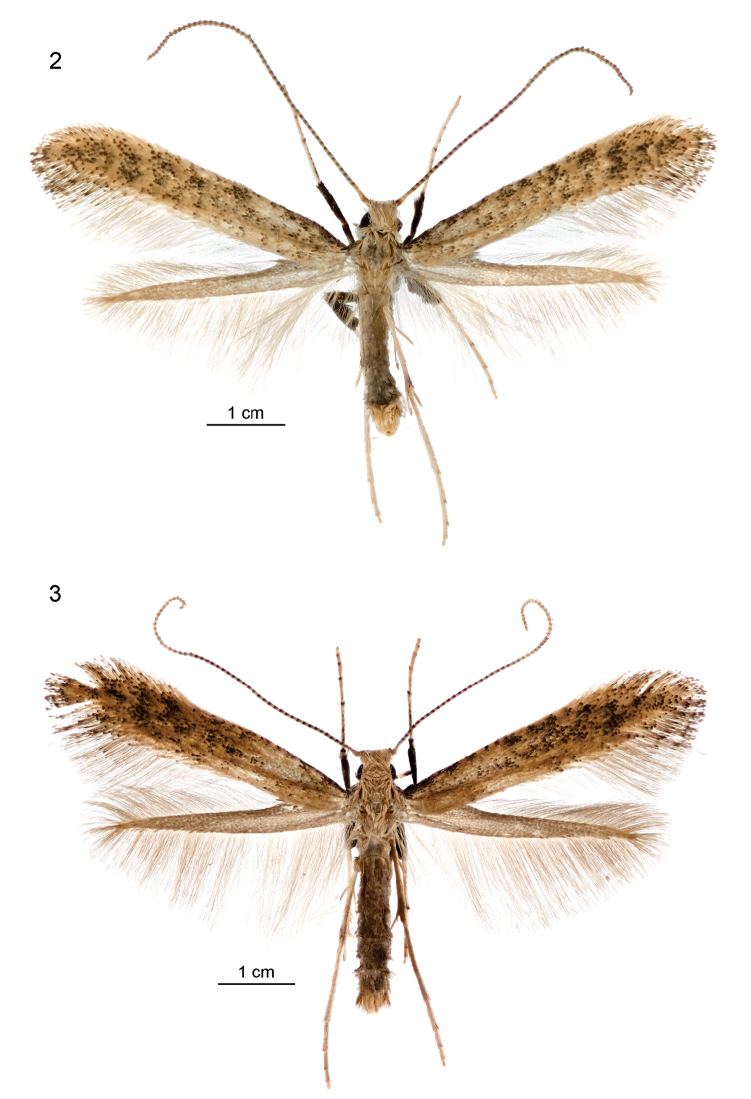
*Sabulopteryxbotanica*, adults. **1** Male paratype, Halswell Quarry (Kennedy’s Bush Rd), Christchurch MC, 29 Mar 2014 (NZAC) **2** female paratype, Christchurch Botanic Gardens MC, emg. 24 Apr 2014 (NZAC).

Wingspan 8.5–10 mm. *Adult male* (Fig. 2): Head: frons white; vertex yellow-ochreous with some scales tipped darker brown; labial palpus whitish with segments 2 and 3 tipped brown; antenna ochreous, ringed dark brownish (apex of each flagellomere), approximately equal in length to forewing; scape with inconspicuous pecten of ca 5 short bristles (often abraded away). Thorax yellow ochreous with tegulae anteriorly blackish. Forewing: yellow-ochreous, with numerous blackish scales forming variable and irregular pattern of broken transverse fasciae; blackish scales often denser towards base of costa and in disc at ca 2/3 length of wing; fringe ochreous whitish, darker around apex to tornus, where dark-tipped scales form three indistinct fringe-lines (in fresh specimens). Hindwing pale greyish; fringe greyish white. Underside: forewing dark brown, paler on dorsum below fold, yellowish around base of cilia; hindwing dark brown on costa and dorsum, pale greyish centrally. Legs: foreleg and midleg with femur and tibia thickened with blackish scales and tarsi yellowish, each tarsomere tipped blackish above; hindleg yellowish, femur with black central patch exteriorly, tibia ringed brownish subapically and each tarsomere with a few brownish apical scales. Abdomen silvery grey, with yellowish anal tuft.

*Adult female* (Fig. 3). As described for male, but abdomen tipped with glossy ochreous whitish scales.

**Figures 4–6.**
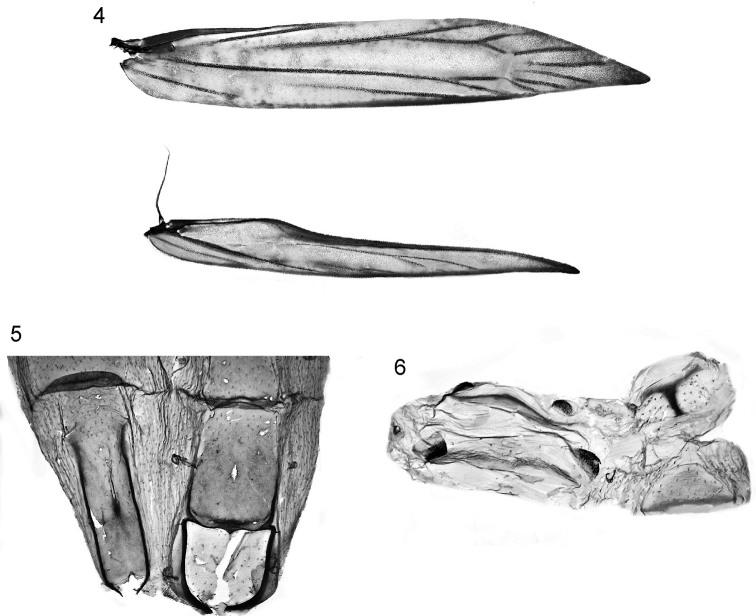
*Sabulopteryxbotanica*, adult morphology. **4** Wing venation **5** male abdominal base **6** male abdomen, segments 7–8 (sternites on left).

Wing venation (Fig. 4). Forewing 12-veined, as described for the genus by Triberti (1985), who regarded the 12-veined condition as being due to coincidence of M2 and M3. Discal cell somewhat dilated posteriorly as described by Triberti (1985). Hindwing very narrow (more so than in other *Sabulopteryx* species) with Rs strongly approximated to costa for most of its length; cell open between M2 and M3; M3 and CuA1 closely approximated and parallel.

**Figures 7–11.**
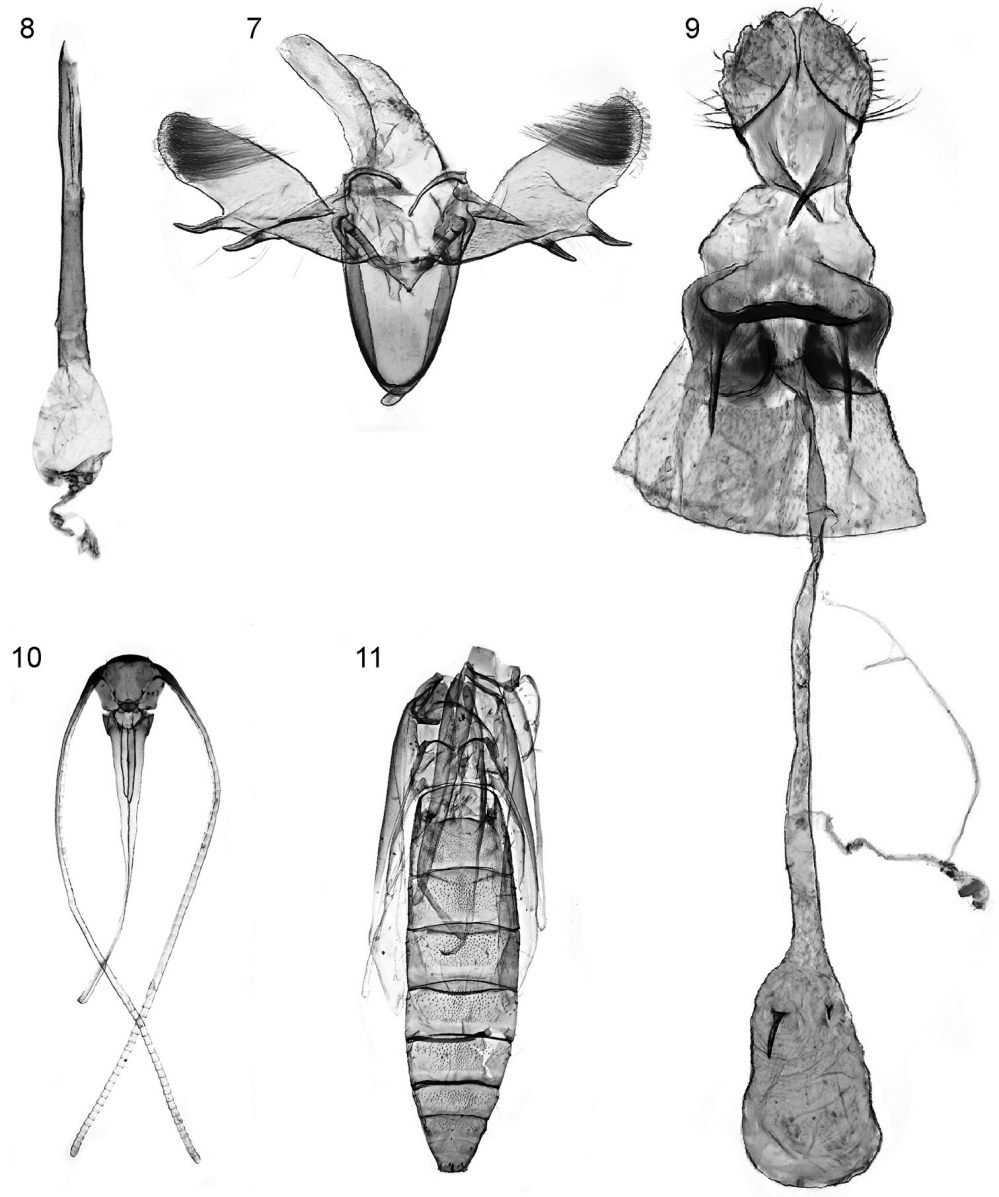
*Sabulopteryxbotanica*, adult and pupal morphology. **7** Male genital capsule **8** male phallus **9** female genitalia **10** pupal exuviae, head, ventral view **11** pupal exuviae, thorax and abdomen, dorsal view.

*Male abdomen and genitalia* (Figs 5–8). Abdominal base as in Fig. 5. S7 and S8 (Fig. 6) markedly shortened and much wider than long, each with lateral pair of coremata. T8 (Fig. 6) with T-shaped sclerite. Genital capsule (Fig. 7): tegumen rather weakly sclerotised, elongate-triangular with attenuate apex. Tuba analis longer than tegumen; subscaphium slender, weakly sclerotised. Valva oblong, narrowed at base, with rounded apex; apical third bearing numerous long fine setae directed obliquely towards costa. Base of valva complex: costa extended into anteriorly-directed narrow, weakly curved process dorsad of anellus membrane (not fused with process from opposite valva, i.e. forming transtilla broken in the middle); from base of this process sclerotised ridge extends across inner (ventral) face of valva to base of long, sclerotised weakly curved spine that extends from valval dorsum at 1/3 valva length; a second, similar spine (slightly more strongly curved) on valval dorsum at 1/2 valva length. Juxta absent. Vinculum large, oval, saccus not differentiated. Phallus (Fig. 8) very elongated, slender, with sharply pointed apex; basally extending smoothly into ovoid bulbus ejaculatorius; vesica without cornuti; caecum penis absent.

*Female genitalia* (Fig. 9). Ovipositor lobes rather short, membranous, basally with long setae, setae denser and shorter towards apex of each lobe; apophyses posteriores short. S7-S8 intersegmental membrane with pair of deep membranous sublateral invaginations; ostium lying between these, dorsal wall of ostium extended into T-shaped membranous area bordered posteriorly by strongly sclerotised transverse lamella postvaginalis, which is continuous with and broadens into lateral sclerotisations of S8. Ductus bursae entirely membranous, long and slender, ca 3× length of corpus bursae; corpus bursae more or less ovoid, with pair of spine-like signa, one long and one very short.

**Figures 12–15.**
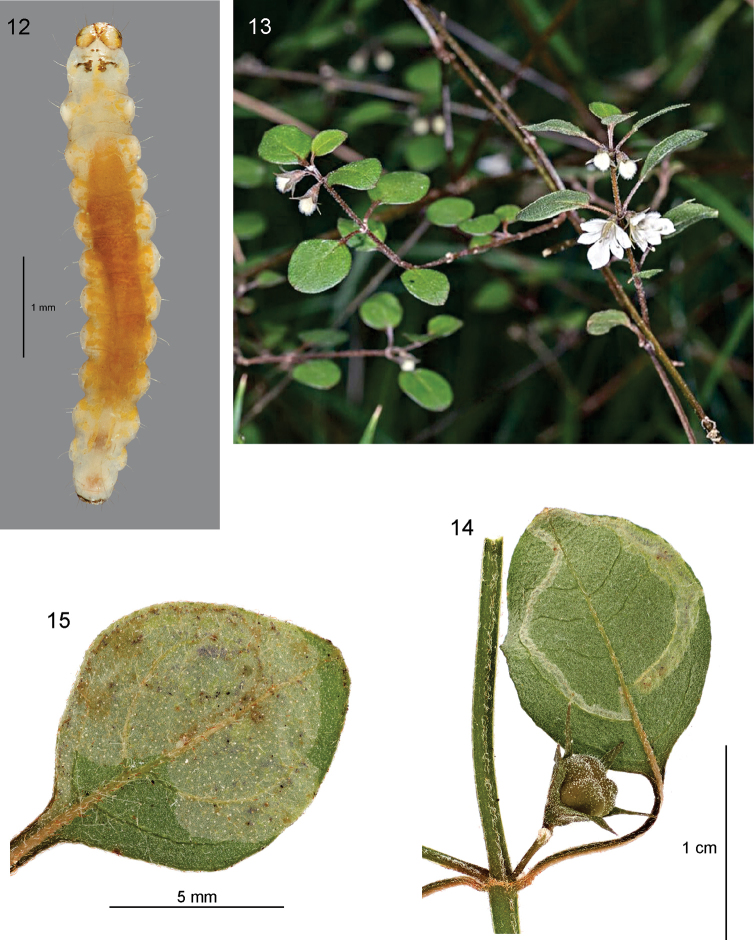
*Sabulopteryxbotanica*, larva, host plant, and early mine. **12** Larva, dorsal **13***Teucriumparvifolium* in flower, Longbush, Wairarapa (photo courtesy of J. Rolfe) **14** initial linear mine on leaf underside **15** early blotch mine.

*Immature stages. Egg.* Elongate-oval, flat, showing up as silvery white translucent ‘shell’ at start of mine, apparently with rather coarsely sculptured chorion (not observed under SEM). *Larva* (Fig. 12). Head translucent pale yellow-brown, margined dark brown posteriorly and along adfrontal / ecdysial lines; blackish in region of stemmata. Body translucent yellowish white, with the gut contents showing through bright green; prothoracic plate in form of two irregular r-shaped sclerites with outlying smaller sclerites anteriorly. Thoracic legs with sclerotised areas dark grey-brown. Prolegs present on A3–5 and A10; crochets on A3–5 biserial: outer row a complete circle, with anterior crochets reduced, inner row a transverse semicircular band of larger crochets in posterior half of planta; A10 with single transverse band of large crochets in anterior half of planta. Anal plate a small transverse brownish sclerite with poorly defined margins. Chaetotaxy as described and figured for *Aspilapteryxtringipennella* (Zeller, 1839) by Triberti (1985). *Pupa*. Head (Fig. 10): frons smoothly rounded, without processes, without setae near antennal bases; antennae ca 3× as long as labial palpi. Thorax (Fig. 11): mesothorax and metathorax each with one pair of well-developed dorsal setae; forewings reaching to ca 1/2 way along A5, hindwings to A3/A4 junction. Abdomen (Fig. 11): A2–8 each with irregular rows of spinules dorsally, spinules slightly smaller and more widely spaced on A2; A7 not furrowed ventrally; abdominal tip truncate, with 3 pairs of small spinose tubercles.

#### Host plant.

The only known host plant is the small-leaved divaricating shrub *Teucriumparvifolium* (Lamiaceae) (Fig. 13), which is endemic to New Zealand and was until recently placed in its own monotypic genus *Teucridium*, and in the family Verbenaceae. Salmaki et al. (2016) showed that *Teucridium* belongs to Lamiaceae and is phylogenetically nested within the large worldwide genus *Teucrium*. The shrub is widespread on both main islands of New Zealand, but rare and very local, and has a conservation status of ‘At Risk – Declining’ (de Lange et al. 2018). The habitat is described as ‘fertile stream sides and river terraces in lowland dry forest and podocarp-hardwood forest, occasionally on forest margins, clearings and amongst scrub’ (NZPCN 2019). These fertile alluvial habitats have been cleared of forest throughout much of the country. Since no other species of *Teucrium* is native to New Zealand, *Sabulopteryxbotanica* must be considered strictly monophagous on *T.parvifolium* (see also Remarks below).

#### Biology.

**Figures 16–18.**
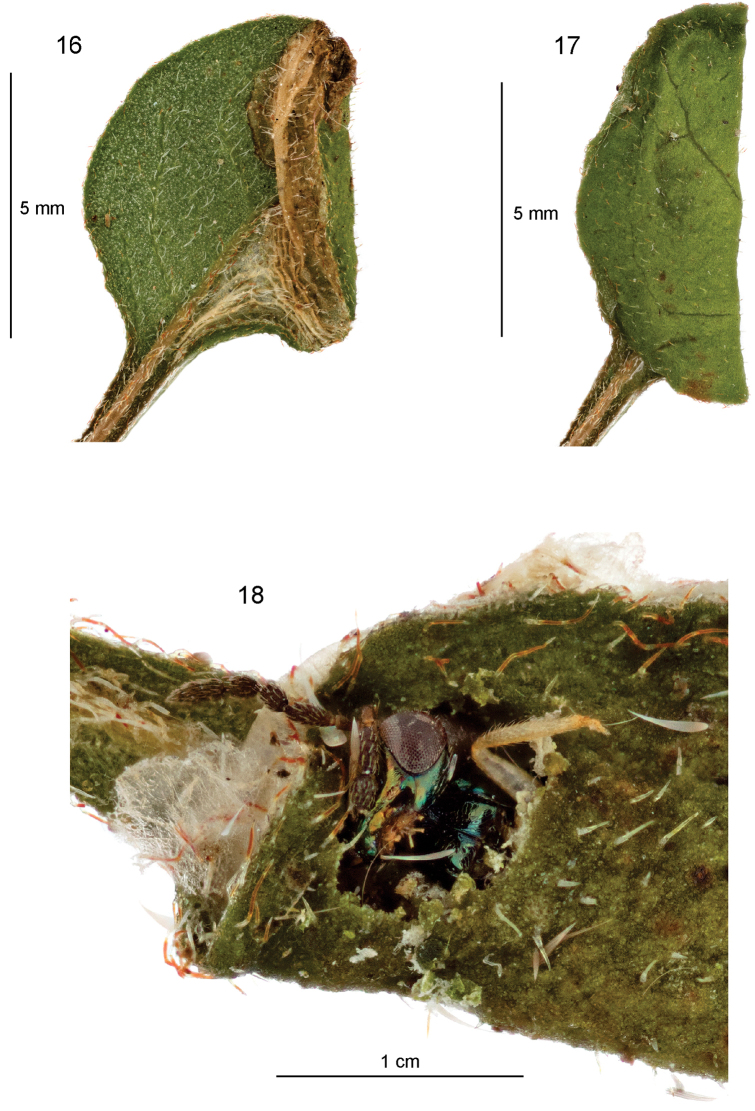
*Sabulopteryxbotanica*, late mine, cocoon, and parasitoid. **16** Fully formed mine with silk spinning causing creases **17** folded leaf with cocoon **18***Sympiesis* sp. (Hymenoptera: Eulophidae) partially emerged from cocoon of *S.botanica* in leaf from herbarium sheet AK285999 (Auckland Museum).

The egg is laid on a leaf of the host plant, usually on the underside next to the midrib. The young sap-feeding larva forms a linear white mine (Fig. 14), almost invariably on the leaf underside, that extends to the margin of the leaf (on the side of the midrib that the egg was laid), then typically crosses the midrib at the leaf apex and extends for some way down the leaf margin on the other side. A line of blackish frass is more or less visible in the centre of the mine at this stage. The larva then doubles back and begins to expand the mine into a white blotch (Fig. 15), usually concentrated towards the leaf apex or to one side of the midrib, but often taking up the whole leaf on smaller leaves. These early mine stages are often rather hard to see unless the leaf is examined closely from the underside. Occasionally the egg and initial mine are on the upperside. Later the larva expands the mine and spins silk extensively in the interior (on the eroded inner surface of the leaf underside), causing the leaf to fold and creating creases in the underside (Fig. 16), in the typical manner of many gracillariid miners. At this stage, patches of the palisade mesophyll are eaten, leaving small windows of upper epidermis towards the middle of the leaf (appearing like holes), and larger windows (browning with age) towards the leaf margin. Black frass is scattered across the inner surface of the upperside of the leaf. When full-fed, the larva leaves the mine and folds a fresh leaf in half from the underside (Fig. 17), forming a cocoon of dense white silk within, in which it pupates. (In captivity, some larvae spin cocoons in tissue paper at the bottom of the rearing container.)

#### Parasitoids.

One species of hymenopteran parasitoid can be confidently associated with the early stages of *S.botanica*, and one tentatively. A specimen of an unidentified species of *Sympiesis* Förster, 1856 (Eulophidae: Eulophinae) was found partially emerged from a cocoon in a folded leaf on an Auckland Museum herbarium sheet (Fig. 18). The plant specimen (AK285999) was collected at Pareora Scenic Reserve SC on 17 Mar 2004 by P.J. de Lange. (The wasp specimen was removed and mounted, and is now in NZAC, cross-referenced with the herbarium sheet.) Interestingly, two species of *Sympiesis* (*S.euspilapterygis* (Erdös, 1958) and *S.gregori* Boucek, 1959) have been associated with the *Teucrium*-mining *Sabulopteryxlimosella* in Europe, but both also attack other leaf-mining Lepidoptera (see references in Noyes 2018, De Prins and De Prins 2018).

One specimen of an unidentified species of *Dolichogenidea* Vierek, 1911 (Braconidae: Microgastrinae) was reared from amongst *Teucrium* leaf-mines collected in Christchurch Botanic Gardens on 23 Jan 2018, emerging on 29 Jan (NZAC). It is thought most likely that this wasp was a parasitoid of *S.botanica*; however, host remains were not found and the sample was discovered later to include one unidentified early instar tortricid larva (preserved, not parasitised). The genus *Dolichogenidea* does not appear to have been associated with *Sabulopteryx* before, but is recorded overseas from other Gracillariinae (*Caloptilia* spp. and *Gracillariasyringella* (Fabricius, 1794)) as well as Lithocolletinae (*Phyllonorycter* spp.), Ornixolinae (*Parectopaononidis* (Zeller, 1839)) (De Prins and De Prins 2018) and many other Lepidoptera, especially microlepidoptera (Austin and Dangerfield 1992). Most reared material of New Zealand *Sympiesis* and *Dolichogenidea* spp. in NZAC is associated with larvae of Tortricidae (Tortricinae).

#### Distribution.

New Zealand, from the following regions: CL, TO, GB, HB, RI, WI, WA / NN, MC, SC, CO.

#### Note.

**Figure 19.**
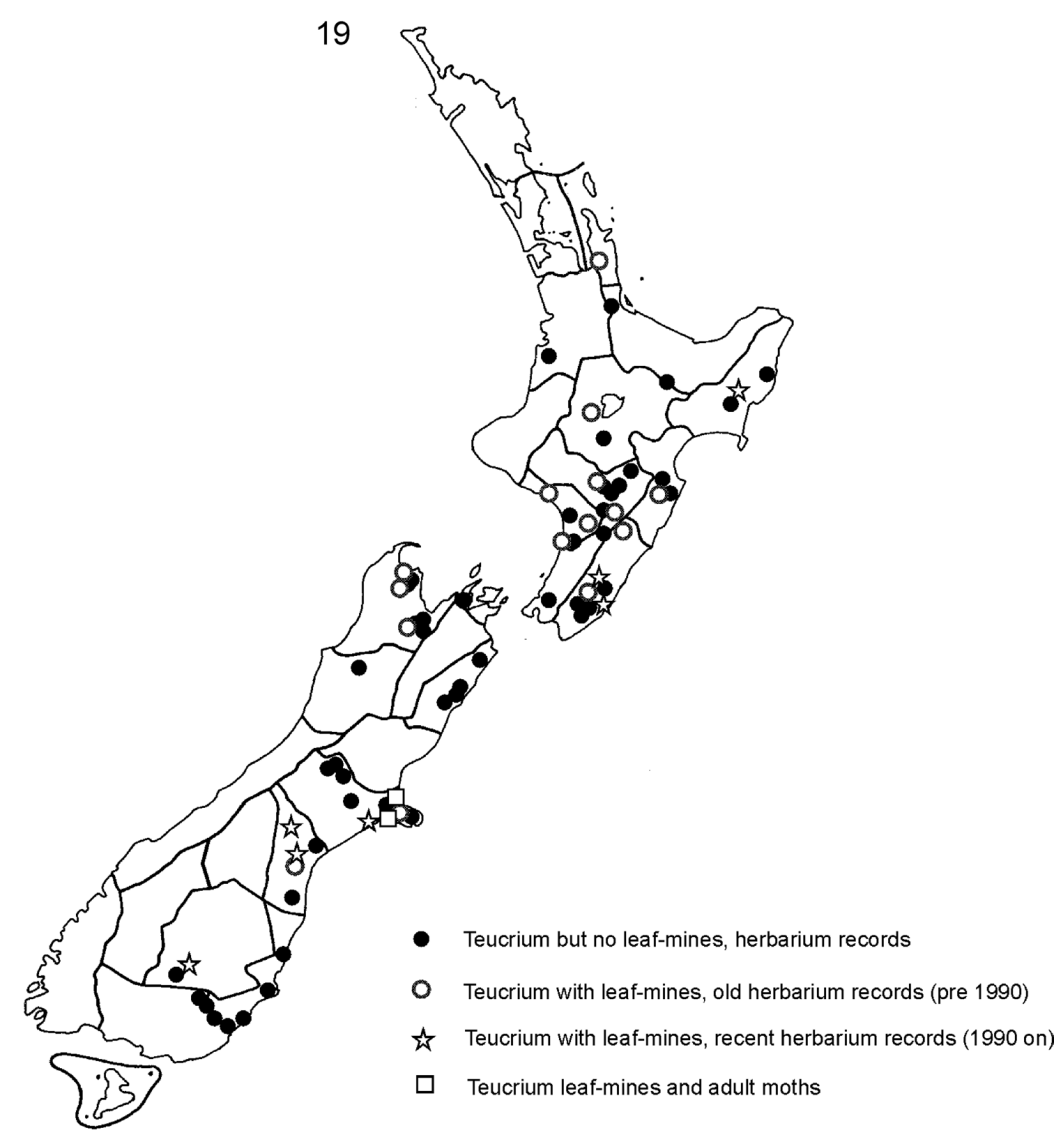
Distribution of *Teucriumparvifolium* and *Sabulopteryxbotanica* based mainly on records from herbarium sheets in Allan Herbarium, Lincoln and Auckland Museum herbarium.

The adult moth has only so far been found or reared in mid Canterbury (MC), but records of leaf-mines and cocoons on herbarium specimens of the host reveal a much wider range (Fig. 19). In some of these areas the plant is very likely to be severely threatened or even extinct, and renewed searches for plant and moth are desirable throughout the country. Towards the north and south of the plant’s range, herbarium records of mines are scarce. The only Coromandel record is from a herbarium specimen collected at Kauaeranga near Thames prior to 1906 by J. Adams (Auckland Museum AK108237); no recent material of the plant from this area was seen. The only Otago record is from Gorge Creek, near Roxburgh CO, where P.N. Johnson found a colony of *Teucrium* in a shaded rock cleft on 24 May 1993 (Allan Herbarium CHR481347; two early mines and one cocoon). It should be noted that only two major herbaria were visited during the course of this research, and there are likely to be preserved mines in other botanical collections that have not yet been visited. The host plant is not known from any offshore islands of New Zealand, so these have been omitted from the map (Fig. 19).

#### Flight period.

Adults have been found in the wild or emerged from wild-collected larvae or pupae in every month of the year in mid Canterbury, and the species is probably more or less continuously brooded here. All stages from early mines to adults can usually be found in the Christchurch Botanic Gardens, where the species is common amongst its host. Phenology in other parts of the range is unknown.

#### Etymology.

The species name refers to the close association of this species with botany and botanists. It was discovered by a botanist (AE Esler) as a pressed larva in a botanical specimen of the host plant, and many further leaf-mines have now been found on herbarium sheets collected as part of botanical surveys. Its discovery by BHP as an adult in the type locality, Christchurch Botanic Gardens, completes the association.

#### Conservation status and potential management.

*Sabulopteryxbotanica* was accorded ‘Nationally Vulnerable’ status in the latest review of the conservation status of New Zealand Lepidoptera, where it was listed as *Caloptilia* sp. “Teucridium” (Hoare et al. 2017). This ranks as the third most critical category assigned to extant species (after ‘Nationally Critical’ and ‘Nationally Endangered’), and was based on the rarity and declining status of the moth’s host plant. As noted above, survey for *S.botanica* through most of the range of its host plant has been inadequate; most herbarium records of mines are over 25 years old (Fig. 19), and further field-work is needed to determine the moth’s current distribution. *Teucriumparvifolium* is an attractive, easily propagated and cultivated shrub that is tolerant of a wide range of conditions (NZPCN 2019), though relatively short-lived (P. Bellingham, pers. comm.). The moth appears to be thriving in situations where *T.parvifolium* has been planted around Christchurch, for example in the Botanical Gardens and in the native plantings maintained by botanist Carol Jensen at Kennedy’s Bush near Halswell Quarry. It should therefore be possible to boost the moth’s population substantially by encouraging the propagation and cultivation of the host plant, with due attention to appropriate sourcing and hygiene. This could be achieved in natural environments where the plant persists, as well as in public plantings and in suburban gardens.

#### Systematic placement: morphology.

The new species described here can be confidently placed in the *Gracillaria* group of genera (Gracillariinae) as defined by Kumata (1982), based especially on the following characters: mid femur and tibia thickened beneath with rough scales; R1 of forewing arising near base of wing, with upper vein of cell weakened beyond branching point of R1; hindwing R2+3 very short and running parallel with and very close to apical part of Sc+R1; hindwing cell open between M2 and M3; male segments 7 and 8 weakly membranous, with coremata. The following genera belong to the *Gracillaria* group, based on Kumata (1982) and updates from subsequent authors (e.g., Triberti 1985, Huemer et al. 2016, Pereira et al. 2019): *Aspilapteryx*, *Caloptilia* Hübner, 1825, *Calybites* Hübner, 1822, *Ectropina* Vári, 1961, *Eucalybites* Kumata, 1982, *Euspilapteryx* Stephens, 1835 (treated as a synonym of *Calybites* by Kumata (1982)), *Gracillaria* Haworth, 1828, *Mercantouria* Huemer, Lopez-Vaamonde & Triberti, 2016, *Povolnya* Kuznetzov, 1979, *Sabulopteryx* and *Vallissiana* Pereira & Arévolo, 2019.

When first discovered as an adult by BHP in January 2013, and before detailed morphological examination, *S.botanica* was tentatively considered to be a member of the genus *Caloptilia* (Hoare et al. 2017: see above). However, RJBH later noted its remarkable external similarity to some west Palaearctic gracillariids then placed in the genus Aspilapteryx (subgenusSabulopteryx), i.e. *S.limosella* from central and southern Europe and *S.inquinata* from southern Europe, Turkey and Lebanon, which it closely resembles in size, wing shape and overall coloration. When describing *Sabulopteryx* as a new subgenus of *Aspilapteryx*, Triberti (1985) anticipated the possibility that it might deserve full genus status. In a recent molecular phylogeny, Pereira et al. (2019) found a 14 to 16% divergence in DNA barcodes between *Aspilapteryx* and *Sabulopteryx* species, and indicated that *Aspilapteryx* is polyphyletic if *Sabulopteryx* is included. Our analysis (see below) also retrieves *Aspilapteryx* and *Sabulopteryx* in separate lineages, so we accept the conclusions of Pereira et al. (2019) and treat *Sabulopteryx* as a genus.

As pointed out by Huemer et al. (2016), morphological comparisons within the *Gracillaria* group are complicated by the apparently homoplasious distribution of character states amongst genera. Based largely on comparison with the descriptions and figures in Vári (1961), Kumata (1982), Triberti (1985), Huemer et al. (2016) and Pereira et al. (2019), the characters listed below in combination lend support for placing the New Zealand *Teucrium*-miner in *Sabulopteryx*:

1. Male abdomen with coremata on both segment 7 and segment 8 (Fig. 6). This conforms with most genera of the *Gracillaria* group, including *Sabulopteryx* and *Mercantouria* (Huemer et al. 2016), but not with *Gracillaria* or *Aspilapteryx*, where there is only one pair of coremata (Kumata 1982; Triberti 1985), nor with *Vallissiana*, where there are no coremata (Pereira et al. 2019).

2. Outline of male valva (beyond sacculus) rounded, not angular, and lacking a ventro-apical lobe. In its rounded / oblong valva, *S.botanica* resembles most genera of the *Gracillaria* group, but not *Mercantouria*, *Calybites* or *Euspilapteryx*, all of which have a distinctly angular valva (Kumata 1982; Huemer et al. 2016), nor *Aspilapteryx* or *Vallissiana*, both of which have a distinct ventro-apical lobe (Pereira et al. 2019).

3. Setae of valva confined to apical area, not extending into basal half. This character does not appear to have been commented on by previous authors: *Caloptilia*, *Gracillaria*, *Povolnya* and *Calybites* all have the valva more extensively setose than the remaining genera of the *Gracillaria* group (including *Sabulopteryx*), perhaps as a result of the relative reduction of the (non-setose) sacculus in these four genera.

4. Valva lacking stout peg-like or spine-like setae distally. This conforms with most genera of the *Gracillaria* group, including *Sabulopteryx*. Short, stout setae are present in the distal part of the valva in *Euspilapteryx* and on the ventrodistal margin in *Calybites* (Kumata 1982), and longer, spine-like setae in *Eucalybites* (Kumata 1982) and *Mercantouria* (Huemer et al. 2016).

5. Male phallus short and straight, without apical processes. The phallus of *S.botanica* (Fig. 8) is similar to those of described species of *Caloptilia*, *Gracillaria*, *Povolnya* and *Sabulopteryx*. It lacks the apical modifications found in *Eucalybites* (Kumata 1982: figs 47 B, C), *Euspilapteryx* and *Vallissiana* (Pereira et al. 2019: fig. 3F) and the rod-like apical sclerite of *Mercantouria* (Huemer et al. 2016: fig. 5). The phallus is long with a helical tip in *Aspilapteryx* (Triberti 1985; Huemer et al. 2016), very long and straight in *Calybites* (Kumata 1982), and curved or sinuous in *Ectropina* (Vári 1961).

6. Female genitalia with two curved, spine-like signa (Fig. 9). This is typical of *Aspilapteryx* and *Sabulopteryx* (Triberti 1985), *Mercantouria* (Huemer et al. 2016), most *Caloptilia* and *Eucalybites* (Kumata 1982), but not of *Gracillaria*, CaloptiliasubgenusMinyoptilia Kumata, 1982, *Calybites*, *Ectropina*, *Euspilapteryx*, or *Vallissiana*, in all of which there is only a single signum (Vári 1961; Kumata 1982; Pereira et al. 2019). *Povolnya* has two signa, but these are short and stout (Kumata 1982).

7. Forewing brownish, without costal streak and with numerous irregularly arranged darker blotches (Figs 1, 2). This wing pattern accords with the description of *Sabulopteryx* by Triberti (1985), and as noted above, there is a strong superficial resemblance between adults of *S.botanica* and the two Palaearctic members of *Sabulopteryx*, *S.limosella* and *S.inquinata*. No other member of the *Gracillaria* group closely approaches this wing pattern.

8. Host-plant genus *Teucrium*. The hostplant genus is shared with *S.limosella*, type species of *Sabulopteryx*, which mines in *Teucriumchamaedrys* L. and *T.montanum* L. in xerothermic localities in central and southern Europe (Triberti 1985). The biology of the two species is also similar. No other gracillariid is known to mine in *Teucrium* (De Prins and De Prins 2018).

In addition, the pupal exuviae of *S.botanica* (Figs 10, 11) match the description and illustrations of the pupa of *Sabulopteryxlimosella* in the key to Gracillariidae pupae by Patočka and Turčáni (2005: 75–76). The exuviae readily key out to *Aspilapteryx* in this key, but since the characters of *Aspilapteryx* were based only on *S.limosella*, the name *Sabulopteryx* should be substituted. Characters of *S.botanica* that lead in this key to *Sabulopteryx* are as follows: proboscis long, exceeding prothoracic femora; head without projection and rounded in lateral view; pronotum not disconnected on dorsomeson; frontal setae absent; A7 without longitudinal furrows ventrally; A10 with spine-like tubercles. From the description and illustrations in Pereira et al. (2019), the pupa of *Vallissianauniversitaria* Pereira & Arévolo, 2019 shares most of these characters with *Sabulopteryx*.

*Sabulopteryxbotanica* differs strongly in some characters from its Palaearctic congeners. Neither of the other species has two large spine-like processes on the male valva (Fig. 7); in *S.limosella* and *S.inquinata* the single process is on or near the valval costa (Triberti 1985: plate VI B, D); *S.botanica* has the processes on the valval dorsum. The placement of the ostium in the female genitalia in *S.botanica* (in the intersegmental membrane between S7 and S8, Fig. 9) is also atypical of *Sabulopteryx*: in the other species it is at the caudal edge of S7 (Triberti 1985). The invaginations of the intersegmental membrane either side of the ostium (Fig. 9) are apparently unique to *S.botanica*. The male of *S.botanica* has T8 in the form of a T-shaped sclerite (Fig. 6), as in genus *Aspilapteryx*. Given the morphological and molecular support (see below) indicating a close relationship between *S.botanica* and the other species of *Sabulopteryx*, these anomalous characters are tentatively considered autapomorphic.

#### Systematic placement: molecular phylogenetics.

**Figure 20.**
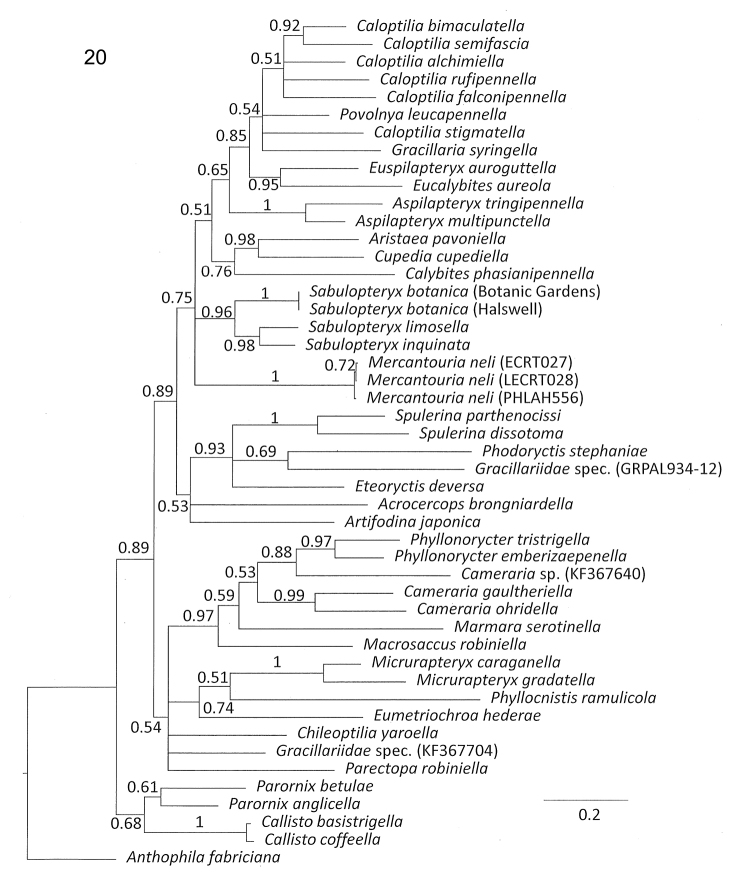
Bayesian consensus phylogeny reconstructed from the COI gene. Numbers above branches represent posterior probabilities. Branch lengths are drawn proportional to the estimated number of substitutions per site, following the scale bar. The tree is rooted with *Anthophilafabriciana* (Choreutidae).

Our molecular analysis, based as it is on a single gene, in no way supplants the much more substantial analysis by Kawahara et al. (2017), but those authors did not include *Sabulopteryx* (or *Aspilapteryx*) in their phylogeny. Our analysis (Fig. 20) provides provisional molecular support for the placement of *Aspilapteryx* and *Sabulopteryx* in Gracillariinae as suggested by the studies of Kumata (1995), Huemer et al. (2016) and Kawahara et al. (2017), and for the placement of *S.botanica* in *Sabulopteryx*, as indicated above from the morphological comparisons.

Despite being only estimated from a single gene, many of the nodes in the phylogeny received posterior probability support values greater than 0.75. Three of the eight subfamilies recovered as monophyletic by Kawahara et al. (2017) are also recovered here, i.e. Gracillariinae (*Caloptilia* to *Mercantouria*, posterior probability 0.75, Fig. 20), Acrocercopinae (*Spulerina* to *Artifodina*, posterior probability 0.53, Fig. 20), and Parornichinae (*Parornix* to *Callisto*, posterior probability 0.68, Fig. 20). Only a single representative each of Marmarinae (*Marmaraserotinella* Busck, 1915), Phyllocnistinae (*Phyllocnistisramulicola* Langmaid & Corley, 2007) and Oecophyllembiinae (*Eumetriochroahederae* Kumata, 1998) was included. Lithocolletinae (*Macrosaccus*, *Cameraria*, *Phyllonorycter*) were recovered as paraphyletic with respect to Marmarinae, while Ornixolinae (*Parectopa*, *Chileoptila*, *Micrurapteryx*) appeared paraphyletic with respect to Oecophyllembiinae + Phyllocnistinae.

The inconsistencies in our cladogram with respect to Kawahara et al. (2017) are to be expected from a single-gene tree, and suggest the limitations of the current analysis with respect to deeper nodes of the phylogeny. Similarly, there are some inconsistencies with the tree recovered by Huemer et al. (2016), but again these are to be expected and do not undermine the evidence for a close relationship between *Sabulopteryxbotanica* and *S.limosella* + *S.inquinata* based on COI, morphology and biology.

The molecular phylogeny supports the treatment of *Aspilapteryx* and *Sabulopteryx* as separate genera (Fig. 20), as proposed by Pereira et al. (2019). The two clades are supported as monophyletic with posterior probabilities of 1 and 0.96 respectively. Though the two clades are separated by two nodes that are only weakly supported (0.51 and 0.65 posterior probabilities, Fig. 20), we consider the evidence from the two independent analyses coupled with the differences in morphology and biology outlined by Triberti (1985) and Pereira et al. (2019) as sufficient to warrant full genus status for *Sabulopteryx*.

#### Remarks.

Lees et al. (2011) nicely demonstrated the importance of herbarium specimens as a source of information on insect-plant interactions. They searched herbaria for preserved mines of the horse-chestnut leaf miner *Camerariaohridella* Deschka & Dimić, 1986 (Gracillariidae: Lithocolletinae), which has recently become invasive throughout Europe, in spite of remaining undetected by entomologists until 1984. From these pressed mines and the larval remains they contained, they were able to document the historical presence of this species in the native range of its host (*Aesculushippocastanum* L.) back to 1879, as well as revealing past outbreaks of the moth and novel haplotypes.

Similarly, study of herbarium material (e.g., Fig. 1), in addition to alerting entomologists to the existence of *Sabulopteryxbotanica*, has produced many historical records of the moth. It has greatly helped our knowledge of the distribution and also provided a parasitoid record (see above). The mines are not difficult to find on herbarium sheets, though sometimes magnification is required to scan for the earliest stages. Of 159 herbarium sheets examined in Auckland and Lincoln, 32 (20%) had at least one leaf-mine of *S.botanica*. The oldest specimen so far found was a single early mine in a leaf from the Cheeseman collection in the Auckland Museum (AK7584): this was collected at Foxhill near Wakefield NN in January 1882. The plant specimen has been annotated appropriately in the Auckland Museum database and the mined leaf is now arrowed on the sheet (E. Cameron, pers. comm.). These old records of the moth also help to confirm that it is an endemic species on its natural host plant and not a recent adventive that has switched to *T.parvifolium* from an introduced *Teucrium* species. To check this assumption further, RJBH examined all New Zealand specimens of introduced species of *Teucrium* (including cultivated species) in the Allan Herbarium in June 2018, and found no evidence of any mines or cocoons. The following species were examined: *Teucriumbetonicum* L’Hér., *T.chamaedrys*, *T.flavum* L., *T.fruticans* L., *T.hircanicum* L., *T.polium* L., *T.pseudochamaepitys* L. and *T.scorodonia* L. Of these, probably only *Teucriumhircanicum* and *T.scorodonia* are established in the wild in New Zealand (NZPCN 2019).

## Discussion

The discovery of an endemic species of *Sabulopteryx* in New Zealand is remarkable and unexpected. The close relationship of *S.botanica* to the European *S.limosella* and *S.inquinata* suggests an extraordinary disjunction in distribution within this group. It would be of great interest to elucidate the age of the split between *S.botanica* and its congeners. According to the phylogenetic analysis and molecular dating of Salmaki et al. (2016), *Teucrium* is estimated to have split from its sister-genus *Rubiteucris* about 15.95 mya and to have begun diversifying around 13.13 mya. Presuming that the last common ancestor of *S.botanica* and *S.limosella* was a *Teucrium*-miner, the proposed age of the host-plant genus is far too young to explain the current known distribution of *Sabulopteryx* as a result of vicariance. If we discount extreme long-distance dispersal, either *Sabulopteryx* is a relictual genus that has contracted from a former much wider distribution, or it has been overlooked or misidentified elsewhere (as it was in New Zealand): both could well be true.

In this regard, two taxa that require further study are *Aspilapteryxtessellata* (Turner, 1940) from eastern Australia and *Caloptiliascutellariella* (Braun, 1923) from eastern North America. *Gracilaria* [sic] *tessellata* was transferred to *Aspilapteryx* by Nielsen and Kumata (1996) without further comment and without indicating to which of the then subgenera (*Aspilapteryx* or *Sabulopteryx*) it might belong. The only specimen of *A.tessellata* in ANIC (a syntype from Ebor, N.S.W.) is missing its abdomen. A second syntype is in the Australian Museum, Sydney; this is incorrectly implied to be the holotype on the Atlas of Living Australia website (ALA 2018). Neither specimen was examined for this paper, but a photograph of the ANIC specimen was seen. In wing pattern, *A.tessellata* does not closely resemble *S.botanica* or the other *Sabulopteryx* species; it has much paler forewings with brownish strigulations and lacks distinct blackish speckling. Turner (1940) gives the forewing ground colour as ‘white’, so the pallid appearance is not due to fading of the specimen. In the Allan Herbarium, there are five specimens of the Australian *Teucriumracemosum*, all collected in South Australia or the Northern Territory; no leaf-mines were found on any of these.

*Caloptiliascutellariella* is a leaf-miner on *Scutellaria* (Braun 1923), which belongs to the same family as *Teucrium* (Lamiaceae), a rare host-plant family amongst Gracillariidae (De Prins and De Prins 2018). Based on COI, *C.scutellariella* was recovered as the sister-species to their new genus *Mercantouria* by Huemer et al. (2016: figs 9, 10), who therefore suggested that it was probably misplaced in *Caloptilia*; these authors did not include *Sabulopteryx* species in their molecular phylogeny. The life history and leaf-mine of *C.scutellariella* appear to be very similar to those of *Sabulopteryxlimosella* and *S.botanica* (see images and text at http://www.microleps.org/Guide/Gracillariidae/Gracillariinae/Caloptilia/index.html), differing from typical *Caloptilia* in that all feeding takes place within the mine, and the larva does not emerge to feed in a rolled or folded leaf, only to pupate. The forewing pattern of *C.scutellariella* could possibly be interpreted as essentially similar to that of *Sabulopteryx* with the area of dark irroration increased so as to obscure the brown ground-colour. The systematic placement of *C.scutellariella* is beyond the scope of this paper and we have not examined specimens.

## Supplementary Material

XML Treatment for Sabulopteryx botanica

## Appendix 1

**Revised checklist of New Zealand Gracillariidae**

This revised checklist places all New Zealand Gracillariidae in the subfamilies defined by Kawahara et al. (2017). We have not followed the newer classification of De Prins et al. (2019) for reasons given in the main text, above.

‘*Acrocercops*’ has been used as a catch-all genus for otherwise unplaced species; since only ‘A.’ leucocyma (Meyrick) can be confirmed as belonging to Acrocercopinae, all other species (except the Australian ‘A.’ laciniella (Meyrick)) are removed from *Acrocercops* and tentatively assigned to other genera that at least belong to the appropriate subfamily. Unpublished notes and drawings by John Dugdale in NZAC (Gracillariidae box-file) have been very helpful in determining the placements adopted here. In most cases, the correct genus placement still needs to be ascertained by further study and some species probably belong to undescribed genera (see note on Oecophyllembiinae below). Therefore these genus placements are qualified with ‘*sensu lato*’ (s.l.). The synonymy remains unchanged from Dugdale (1988) and is not repeated here. Brief notes on host plant and biology are given for all species.

E = endemic to New Zealand. A = adventive in New Zealand.

**
Gracillariidae
**

**
Acrocercopinae
**

Acrocercops(s.l.)laciniella (Meyrick, 1880) A. Leaf-miner on juvenile leaves of *Eucalyptus* spp. (Myrtaceae) (Common 1990). Tentatively retained here in Acrocercopinae in the absence of conflicting evidence.

Acrocercops(s.l.)leucocyma (Meyrick, 1889) E. Leaf-miner on *Agathisaustralis* (Araucariaceae), overwintering in petiole galls (Wise 1952; reared material in NZAC). The expanded A8 of the male (J.S. Dugdale, NZAC notes) confirms placement in Acrocercopinae.

*Dialecticascalariella* (Zeller, 1850) A. Leaf-miner on Boraginaceae, including *Echium* spp., *Myosotis* spp. and occasionally *Myosotidiumhortensia* (Chatham Island forget-me-not) (reared material in NZAC).

**
Gracillariinae
**

*Caloptiliaazaleella* (Brants, 1913) A. Leaf-miner and folder on azaleas (*Rhododendron* spp., Ericaceae).

*Caloptiliachalcodelta* (Meyrick, 1889) E. Leaf-miner and folder on *Nestegis* (Oleaceae) (reared material in NZAC).

*Caloptiliachrysitis* (Felder & Rogenhofer, 1875) E. Leaf-miner and folder on *Weinmannia* (Cunoniaceae), *Elaeocarpus* (Elaeocarpaceae) and rarely *Knightiaexcelsa* (Proteaceae) (reared material in NZAC).

*Caloptiliaelaeas* (Meyrick, 1911) E. Leaf-miner and folder on *Coriariaplumosa* and probably other small-leaved *Coriaria* spp. (Coriariaceae) (reared material in NZAC).

*Caloptilialinearis* (Butler, 1877) E. Leaf-miner and folder on *Coriariaarborea* (Coriariaceae) (reared material in NZAC).

*Caloptiliaselenitis* (Meyrick, 1909) E. Leaf-miner on *Lophozoniamenziesii* (Nothofagaceae); cocoon between joined leaves (Watt 1924; reared material in NZAC).

*Macarostolaida* (Meyrick, 1880) A. Leaf-miner and folder on *Eucalyptus* spp. (Myrtaceae). An Australian species found established locally in east Auckland and Northland in January 2019: adults and numerous larvae. New to New Zealand.

*Macarostolaminiella* (Felder & Rogenhofer, 1875) E. Leaf-miner and folder on *Syzygiummaire* (Myrtaceae) (reared material in NZAC).

*Sabulopteryxbotanica* Hoare & Patrick, 2019 E. Leaf-miner and folder on *Teucriumparvifolium* (Lamiaceae) (this paper).

**
Lithocolletinae
**

*Phyllonoryctermessaniella* (Zeller, 1846) A. Leaf-miner on *Quercus* spp. (including deciduous species as well as evergreen *Q.ilex* L.) (Fagaceae) and occasionally *Fagussylvatica* L. (Fagaceae), *Castaneasativa* Mill. (Fagaceae), *Betulapendula* Roth (Betulaceae), *Carpinusbetulus* L. (Corylaceae), *Maluspumila* Mill. (= *M.* x *domestica*) (Rosaceae), *Accasellowiana* (O. Berg) Burret (Myrtaceae) (Wise 1953, 1954). Other New Zealand host plants are also listed by Wise (1953, 1954) based on mines, but only those from which moths were reared are given here.

*Porphyroselahardenbergiella* (Wise, 1957) A. Leaf-miner on *Hardenbergia* (Fabaceae) (Wise 1957). Note. This species has not been collected since 1955, and is still not known from Australia, which is almost certainly its country of origin (*Hardenbergia* is endemic to Australia).

**
Oecophyllembiinae
**

Note. In this subfamily, species feeding on Araliaceae and Apocynaceae are provisionally assigned to *Eumetriochroa* and the single Rubiaceae-miner is assigned to *Corythoxestis*. These provisional assignments need checking, but are considered for the time being more informative and less misleading than the placement of all species in *Acrocercops*. Male genitalia and wing characters (J.S. Dugdale, NZAC notes) as well as leaf-mining biology and pupal characters (Watt 1920) confirm placement in Oecophyllembiinae, but suggest that all these species may turn out to belong to endemic genera, and further study is required.

Eumetriochroa(s.l.)aellomacha (Meyrick, 1880) comb. nov. E. Leaf-miner on *Pseudopanaxarboreus* (Watt 1920). Note. Identification of Meyrick’s species with subsequently reared material follows Watt (1920) but requires checking.

Eumetriochroa(s.l.)aethalota (Meyrick, 1880) comb. nov. E. Leaf-miner and stem-miner on *Parsonsia* (Apocynaceae).

Eumetriochroa(s.l.)panacicorticis (Watt, 1920) comb. nov. E. Stem-miner on *Pseudopanaxarboreus* (Araliaceae) (Watt 1920; reared material in NZAC).

Eumetriochroa(s.l.)panacifinens (Watt, 1920) comb. nov. E. Leaf-miner on *Pseudopanaxarboreus* and probably *P.colensoi* (Araliaceae) (Watt 1920; reared material in NZAC).

Eumetriochroa(s.l.)panacitorsens (Watt, 1920) comb. nov. E. Leaf-miner (leaf underside) on *Pseudopanaxarboreus* and *Raukauasimplex* (Araliaceae) (Watt 1920; reared material in NZAC).

Eumetriochroa(s.l.)panacivagans (Watt, 1920) comb. nov. E. Leaf-miner on *Pseudopanaxcrassifolius* and *P.lessonii* (Araliaceae) (Watt 1920; reared material in NZAC).

Eumetriochroa(s.l.)panacivermiforma (Watt, 1920) comb. nov. E. Leaf-miner on *Raukauaedgerleyi* and *R.simplex* (Araliaceae) (Watt 1920; reared material in NZAC).

Corythoxestis(s.l.)zorionella (Hudson, 1918) comb. nov. E. Leaf-miner on large-leaved *Coprosma* spp. and sometimes *C.arborea* (Rubiaceae) (Watt 1920; reared material in NZAC).

**
Ornixolinae
**

Parectopa(s.l.)alysidota (Meyrick, 1880) comb. nov. A. Phyllode-miner and sometimes stem-miner on *Acacia* spp. (Mimosaceae) (Watt 1920; reared material in NZAC). Note. Based on wing venation, this species is related to *Parectopa* (where it was placed by Meyrick, as *P.citharoda* Meyrick, a synonym). The adult resting posture, with the first two pairs of legs spread wide but appressed to each other, resembles that of other genera of Ornixolinae (e.g., *Epicephala* Meyrick, *Cuphodes* Meyrick (Kawahara et al. 2017: fig. 5H). It may require a new genus (J.S. Dugdale, NZAC notes).

*Conopomorphacyanospila* Meyrick, 1885 E. Fruit-borer on *Alectryonexcelsus* (Sapindaceae) (reared material in NZAC).

*Polysomaeumetalla* (Meyrick, 1880) A. Miner in surface of *Uromycladium* rust galls on *Acacia* (Mimosaceae) (Common 1990; reared material in NZAC).
